# Inflammation, non-endothelial dependent coronary microvascular function and diastolic function—Are they linked?

**DOI:** 10.1371/journal.pone.0236035

**Published:** 2020-07-16

**Authors:** Hannah E. Suhrs, Jakob Schroder, Kira B. Bové, Naja D. Mygind, Daria Frestad, Marie M. Michelsen, Theis Lange, Ida Gustafsson, Jens Kastrup, Eva Prescott

**Affiliations:** 1 Department of Cardiology, Bispebjerg Hospital, University of Copenhagen, Copenhagen, Denmark; 2 Department of Cardiology, Rigshospitalet, University of Copenhagen, Copenhagen, Denmark; 3 Department of Cardiology, Hvidovre Hospital, University of Copenhagen, Copenhagen, Denmark; 4 Department of Public Health, Section of Biostatistics, University of Copenhagen, Copenhagen, Denmark; 5 Center for Statistical Science, Peking University, Beijing, China; Universita degli Studi Magna Graecia di Catanzaro, ITALY

## Abstract

**Purpose:**

Systemic inflammation and coronary microvascular dysfunction (CMD) may be causal drivers of heart failure with preserved ejection fraction (HFpEF). We tested the hypothesis that subclinical inflammation is associated with non-endothelial dependent CMD and diastolic dysfunction.

**Methods:**

In a cross-sectional study of 336 women with angina but no flow limiting coronary artery stenosis (180 with diabetes) and 95 asymptomatic controls, blood samples were analysed for 90 biomarkers of which 34 were part of inflammatory pathways. CMD was assessed as coronary flow velocity reserve (CFVR) by transthoracic Doppler echocardiography and defined as CFVR<2.5. We used E/e’ as an indicator of diastolic function in age-adjusted linear regressions to assess correlations between biomarkers, CFVR and diastolic function.

**Results:**

CMD was found in 59% of participants whereas only 4% fulfilled strict criteria for diastolic dysfunction. Thirty-five biomarkers, 17 of them inflammatory, were negatively correlated with CFVR and 25, 15 inflammatory, were positively correlated with E/e’. A total of 13 biomarkers, 9 inflammatory, were associated with both CFVR and E/e’. CFVR and E/e’ were only correlated in the subgroup of patients with CMD and signs of increased filling pressure (E/e’>10) (p = 0.012).

**Conclusion:**

This is the first study to link a large number of mainly inflammatory biomarkers to both CMD and E/e’, thus confirming a role of inflammation in both conditions. However, despite a high prevalence of CMD, few patients had diastolic dysfunction and the data do not support a major pathophysiologic role of non-endothelial dependent CMD in diastolic dysfunction.

## 1 Introduction

Coronary microvascular dysfunction (CMD) is associated with increased mortality, even in the absence of macrovascular coronary artery disease (CAD) [1, 2]. Among patients with heart failure with preserved ejection fraction (HFpEF) the prevalence of CMD was recently found to be high [3] and it has been suggested that CMD plays a pathophysiological role in the development [4]. Multiple comorbidities including diabetes, hypertension and dyslipidemia contribute to a pro-inflammatory state that induces oxidative stress in the microvascular endothelium, leading to both endothelial dependent or non-endothelial dependent CMD [4, 5]. Because of cross-talk between endothelial cells and cardiomyocytes, endothelial inflammation may ultimately lead to myocardial functional alterations [6–8], contributing to adverse cardiac prognosis [9, 10]. The same comorbidities mentioned above that have been associated with microvascular dysfunction have been associated with cardiac diastolic dysfunction [11, 12]. Further, it has been suggested that angina with no obstructive CAD is pathophysiologically linked to HFpEF [13], but evidence supporting a direct pathophysiological link between CMD and cardiac diastolic dysfunction remains limited [14].

**Fig 1:**
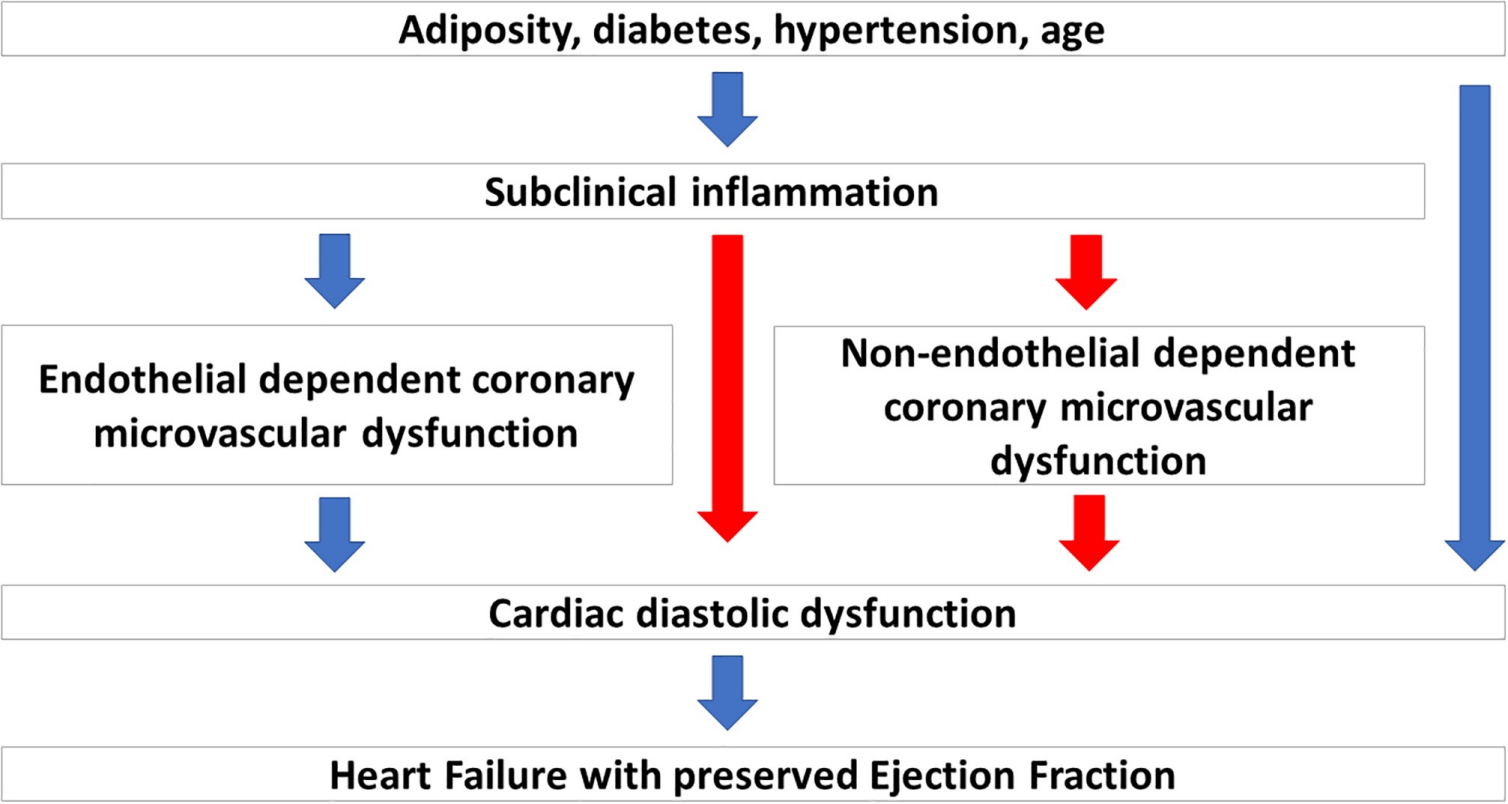
Hypothetical pathway from inflammation to diastolic dysfunction. The underlying hypothesis linking inflammation, coronary microvascular function and diastolic dysfunction through causal pathways. The pathways explored in the current paper are depicted with red arrows.

In smaller studies we have previously found several biomarkers of inflammation and vascular dysfunction to be associated with non-endothelial dependent CMD in women with angina symptoms but no flow limiting CAD [15, 16]. Here we explore the hypothesis that subclinical inflammation is linked to coronary microvascular function and cardiac diastolic function, and that the effect of inflammation on diastolic function is partly mediated by non-endothelial dependent CMD (Fig 1).

## 2 Methods

### 2.1 Population

We explored this hypothesis in a population of diabetic (Group A) and non-diabetic (Group B) women with angina and no flow limiting CAD from the iPOWER (ImProve diagnOsis and treatment of Women with angina pEctoris and micRovessel disease) study as well as in controls (Group C). The iPOWER study has been described in detail previously [17, 18] and comprises 1830 women with angina symptoms and no obstructive CAD defined as <50% coronary artery stenosis assessed by clinically indicated invasive coronary angiogram in eastern Denmark between March 2012 and December 2017. The control group consisted of asymptomatic women without diabetes or previous cardiovascular disease (Group C) recruited from the Copenhagen City Heart Study, a population based prospective study [19]. Patients with diabetes and asymptomatic controls were chosen to achieve a broad spectre of microvascular and diastolic function. Therefore, samples were not matched on baseline characteristics.

The iPOWER study was performed in accordance with the Helsinki Declaration and was approved by the Danish Regional Committee on Biomedical Research Ethics (H-3-2012-005). All participants gave written informed consent on oral and written information.

### 2.2 Examinations

Assessments included demographics, risk factors, clinical data (age, body mass index (BMI), hypertension, hypercholesterolemia, smoking, and medication) and a fasting blood sample analysis.

Participants underwent a standard resting transthoracic echocardiography with measurements of systolic and diastolic function using GE Healthcare Vivid E9 cardiovascular ultrasound system (GE Healthcare, Horten, Norway) with a 1.3–4.0 MHz transducer (GE Vivid 5S probe). Images were stored for off-line analysis (GE EchoPAC v.112, Norway). Please see S1 Data in S1 File: Echocardiographic examination, for a description of the evaluation of diastolic function. We used E/e’ as an indicator of diastolic function in linear regression analyses as has been done in other studies [20, 21].

Microvascular function was assessed by transthoracic Doppler echocardiography (TTDE) measuring coronary flow velocity (CFV) of the left anterior descending artery (LAD) during rest and dipyridamole infusion (0.84 mg/kg) over 6 minutes using a 2.7–8 MHz transducer (GE Vivid 6S probe) as previously described [18, 22]. Coronary flow velocity reserve (CFVR), was calculated as the ratio of peak diastolic CFV during dipyridamole induced hyperemia and rest. Please see S1 Data in S1 File: Echocardiographic examination, for a thorough description of the echocardiographic examinations.

### 2.3 Protein biomarkers

Blood samples were analyzed by Olink Proteomics, Uppsala, Sweden using the predefined cardiovascular disease panel III, measuring 90 protein biomarkers related to the cardiovascular system by real-time polymerase chain reaction. For further description of the method please see S2 Data in S1 File: Biomarker analysis and intensity normalization. It is a well-designed panel of which 34 of 90 biomarkers are classified by the provider as inflammatory, but of the remaining, many are poorly characterized and may also be involved in inflammatory processes. The panel has been used by other investigators, including HFpEF studies [20] and is therefore interesting to explore further.

### 2.4 Statistical analysis

Differences in baseline patient characteristics across groups were analyzed for linear trends by linear regression for continuous outcome variables. Variables with non-Gaussian distribution were naturally log transformed before the analysis which resulted in an approximated Gaussian distribution. Logistic regression analysis was used for categorical outcome variables. Level of biomarker, E/e’ (as an indicator of diastolic function) and CFVR were compared across groups by trend test in age adjusted linear regression.

Since a previous study found association between CMD and E/e’ only in a subgroup with impaired CFVR and E/e’ [23] separate additional regression analyses were performed in a subpopulation with CMD defined as CFVR<2.5 and signs of diastolic impairment defined as E/e’>10. Cut-offs were chosen to include subjects with poor microvascular function and signs of diastolic impairment while keeping the population size sufficiently large for further analyses. In this sub-analysis, three outliers were found to be influential. After values were checked for correctness we found they could not be removed from the dataset, and we examined the data with a robust regression analysis including information on leverage and residual of the observations.

Initial exploratory analyses were performed investigating the relation between each of the 90 biomarkers and CFVR and E/e’, respectively, in regression models as explained above, adjusted for age. To ease comparison across biomarkers, values were transformed to z-scores. We did not perform Bonferroni correction, since we considered the analysis exploratory and our aim was not to identify a single or a few explanatory biomarkers, but to explore possible associations suggestive of an effect of inflammation.

We used a principal component analysis (PCA) to examine biomarker interrelations in the 34 biomarkers defined by the provider as mainly linked to inflammation. Our intention with the PCA was not to reduce data to fewer explanatory components, but to explore the communality within the biomarkers. All 34 inflammatory biomarkers were highly correlated, and all loaded principally and equally on the first component in the PCA. Therefore, we defined a common inflammation index as the mean level of the 34 inflammatory biomarkers. To explore whether CFVR may mediate a causal effect between inflammation and diastolic dysfunction, we performed a mediation analysis using this inflammation index in predicting E/e’ through CFVR in the subgroup defined above (CFVR<2.5 and E/e’>10) (Fig 1). The mediation analysis can formally assess if any association between inflammation and E/e’ is created (ie. mediated) by an effect of inflammation on CFVR, which then in turn affects E/e’. A detailed description of mediation analysis and the mathematical background can be found in Lange et al [24] and is implemented using the R package medflex [25, 26].

Continuous variables with a Gaussian distribution are expressed as mean ± standard deviation (SD) or mean-max values, and continuous variables with a non-Gaussian distribution as appropriate. A two-sided p-value below 0.05 was considered significant. All analyses were performed using STATA/IC 13.1 (StataCorp LP, College Station, Texas, USA), except for the mediation analysis which was performed using the R package medflex.

## 3 Results

After excluding participants without CFVR examination (n = 18) or poor quality CFVR measurement (n = 20) we included 180 women with a diagnosis of diabetes and 156 women without diabetes from the iPOWER cohort [17]. From the Copenhagen City Heart Study cohort, we included a control sample of 95 women [19]. Baseline characteristics are presented in Table 1.

**Table 1:** Population characteristics across the three groups.

	Women with angina and no obstructive CAD		Controls	
	Group A	Group B	Group C	
	Diabetes (n = 180)	No diabetes (n = 156)	Control (n = 95)	p^†^
**Clinical data (mean(SD) / median(IQR) / n (%))**				
Age, years	64 (36,80)	62 (32,80)	61 (32,81)*	0.003
BMI	30.7 (15.2, 45.5)	26.1 (18.6,40.0)*	24.7 (18.6,38.4)*	<0.001
BP, mmHg	135 (23)	129 (19)*	116 (17)*	<0.001
HR at rest, beats/min	73 (12)	66 (19)*	68 (13)*	<0.001
Hypertension	133 (75)	85 (55)*	15 (18)*	<0.001
Ever-smoker	78 (44)	99 (64)*	50 (58)*	0.007
Hypercholesterolemia	145 (82)	98 (63)*	15 (18)*	<0.001
Atheromatosis (on CAG)	83 (49)	54 (35)*		0.244
Postmenopausal	151 (84)	129 (83)	74 (78)	
**Medication (n (%))**				
ASA	105 (59)	67 (43)*	3 (3)*	<0.001
BB	60 (34)	48 (31)	3 (3)*	<0.001
ACE-I	45 (26)	24 (16)*	4 (4)*	<0.001
ANGII-blocker	62 (35)	24 (16)*	6 (6)*	<0.001
Statin	135 (76)	73 (47)*	6 (6)*	<0.001
**Biochemical measures (median (IQR))**				
HbA1c, mmol/mol	50 (45, 61)	37 (34, 39)*	35 (34, 38)*	<0.001
Total cholesterol, mmol/L	4.2 (3.7, 4.8)	5.0 (4.3, 5.7)*	5.3 (4.9, 5.8)*	<0.001
HDL-cholesterol, mmol/L	1.4 (1.1, 1.7)	1.7 (1.4, 2)*	1.8 (1.6, 2.2)*	<0.001
LDL-cholesterol, mmol/L	2 (1.5, 2.6)	2.7 (2, 3.7)*	2.9 (2.5, 3.5)*	<0.001
Triglyceride, mmol/L	1.4 (1, 2)	1.0 (0.8, 1.4)*	0.9 (0.7, 1.1)*	<0.001
Creatinine, μmol/L	62 (55, 71)	65 (58, 74)	67 (61, 74)*	0.030
U-albumin/creatinin ratio	1.3 (0.7, 3.2)	1.0 (0.8, 1.4)*	0.5 (0.3, 1.2)*	<0.001

^†^ Unadjusted trend test from linear or logistic regression.

*p<0.05 compared with group A.

CAG coronary angionagram, CAD coronary artery disease, CVD cardiovascular disease, BMI body mass index, BP blood pressure, HR heart rate, ASA acetyl salicylate, BB beta blocker, ACE-I angiotensin converting enzyme inhibitor, ANGII angiotensin II receptor.

Women with diabetes were older and, as expected, had a higher burden of risk factors compared with non-diabetic patients with and without angina symptoms. Furthermore, they were more likely to have diffuse atheromatosis than non-diabetic angina patients. There was a clear trend of more medical treatment in patients with angina which was also reflected in biochemical measurements of cholesterol levels. Patients with diabetes had well-regulated blood sugar with median (IQR) Hba1c of 50 (45, 61) mmol/l.

### 3.1 CFVR

Median CFVR (IQR) ranged from 2.22 (1.90, 2.56) in the diabetes group to 2.35 (1.96, 2.74) in the symptomatic non-diabetes group and 2.63 (2.22, 3.08) in controls (p for trend <0.001) (Table 2).

**Table 2:** Echocardiographic measures compared across the three groups.

	Women with angina and no obstructive CAD		Controls	
Measure, Mean (SD) / median (IQR)	Group A	Group B	Group C	
	Diabetes (n = 180)	No diabetes (n = 156)	Control (n = 95)	p for trend
CFVR	2.22 (1.90, 2.56)	2.35 (1.96, 2.74)	2.63 (2.22, 3.08)*	<0.001
CFV at rest, m/s	0.24 (0.20, 0.31)	0.23 (0.19, 0.29)	0.21 (0.19, 0.25)*	0.002
CFV at hyperemia, m/s	0.55 (0.45, 0.63)	0.56 (0.47, 0.68)	0.57 (0.49, 0.66)	0.290
GLS at rest, %	20.32 (2.73)	21.28 (3.24)*	21.09 (2.75)	0.018
E/A ratio	0.91 (0.78, 1.05)	0.98 (0.83, 1.13)	1.11 (0.94, 1.33)*	<0.001
e’ (lateral and septal), cm/s	7.89 (2.16)	9.05 (2.28)*	9.49 (2.32)*	<0.001
E/e’ ratio	9.79 (8.14, 12.21)	8.07 (6.85, 9.95)*	7.86 (6.89, 9.58)*	<0.001
LAI, mL/m^2^	26.91 (21.36, 32.36)	26.44 (22.20, 34.15)	22.08 (19.30, 28.06)*	<0.001
LVMI, g/m^2^	71.28 (61.70, 80.70)	73.01 (62.29, 81.46)	75.53 (65.09, 84.55)	0.023
LV mass, g	132.79 (116.07, 155.43)	129.38 (110.02, 150.57)	139.55 (114.68, 158.30)	0.811
DECT, ms	192 (165, 225)	179 (156, 210)	193 (179, 213)	0.522

^†^ Unadjusted trend test from linear or logistic regression.

*p<0.05 compared with group A.

CFVR coronary flow velocity reserve, CFV coronary flow velocity, GLS global longitudinal strain measured as peak systolic strain, LAI left atrial volume indexed by body surface area, LVMI left ventricular myocardial index, DECT deceleration time.

Results did not change after adjusting for age and heart rate at rest. CFV at hyperemia was similar between groups and the lower CFVR was mainly due to higher baseline CFV in the angina patients. The proportion with CMD defined as CFVR<2.5 was 69% in group A, 58% in group B and 38% in group C (p<0.001). The proportion of participants with severe CMD (CFVR<2) was 34% in group A, 27% in group B and 14% in group C.

### 3.2 Systolic and diastolic cardiac function

Several indices of systolic and diastolic function showed a significant trend of poorer function in patients with diabetes compared with nondiabetic women with and without angina symptoms. However, only 4% fulfilled strict criteria for diastolic dysfunction (Please see S1 Data in S1 File: Echocardiographic examination, for the definition). GLS was significantly lower among patients with diabetes whereas E/A ratio, e’, E/e’ and LAI all pointed towards more impairment of diastolic function across groups (p all<0.001). No trend was seen for deceleration time (DECT) and LVMI showed a weak inverse trend (p = 0.02). However, the absolute LV mass did not differ significantly between groups.

### 3.3 Biomarkers

**Fig 2:**
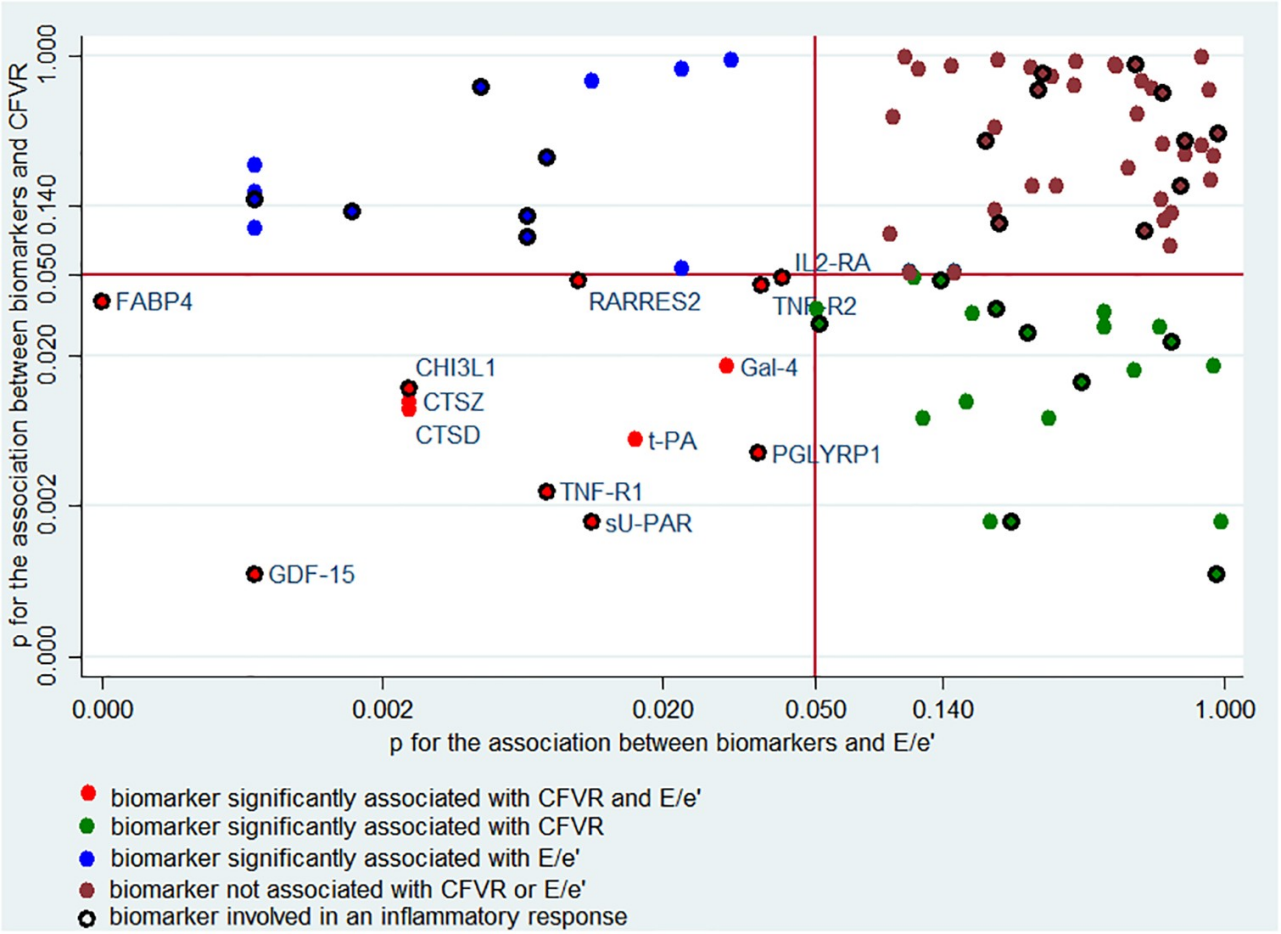
Overview of biomarkers associated with CFVR and E/e’ by level of significance. The red line dividing the graph in four squares marks a significance level of 0.05 on both axes. Lower left quadrant shows the 13 biomarkers significantly associated with both CFVR and E/e’ with inflammatory biomarkers marked with open circles. Biomarkers significantly associated with CFVR in the upper left corner (green dots) and biomarkers significantly associated with E/e’ in the lower right corner (blue dots).

Biomarkers were available in 395 study participants. All 90 biomarkers passed quality control criteria and >95% of the measured proteins were detected in all samples. After age adjustment, 34 biomarkers were significantly associated with CFVR, and all had a negative association, i.e. a higher CFVR was associated with a lower level of biomarkers. Of those, 17 were part of the 34 biomarkers in the inflammatory panel (referring to the pathway from inflammation through non-endothelial dependent coronary microvascular dysfunction to diastolic dysfunction in Fig 1). 26 biomarkers were significantly associated with E/e’, 25 with a positive association, and 15 were in the inflammatory panel (referring to the pathway from inflammation to cardiac diastolic dysfunction depicted in Fig 1). There was an overlap of thirteen biomarkers, significantly associated with both CFVR and E/e’ with 9 being part of the inflammatory panel (please see Fig 2 and S3A Table in S1 File: Biomarker correlations with CFVR and E/e’ after age-adjustment).

The PCA indicated high communality between the inflammatory biomarkers. The combined inflammation score showed significant correlation with both CFVR and E/e’ after age adjustment (p = 0.013 and 0.019, respectively).

Levels of biomarkers across groups A, B and C are shown in S4A Table in S1 File: Level of biomarker across study groups. Of the 47 biomarkers associated with CFVR and/or E/e’, 41 showed a significant negative trend across the groups and no significant trend was found in the remaining 6 of these biomarkers.

#### CMD and diastolic function

**Fig 3:**
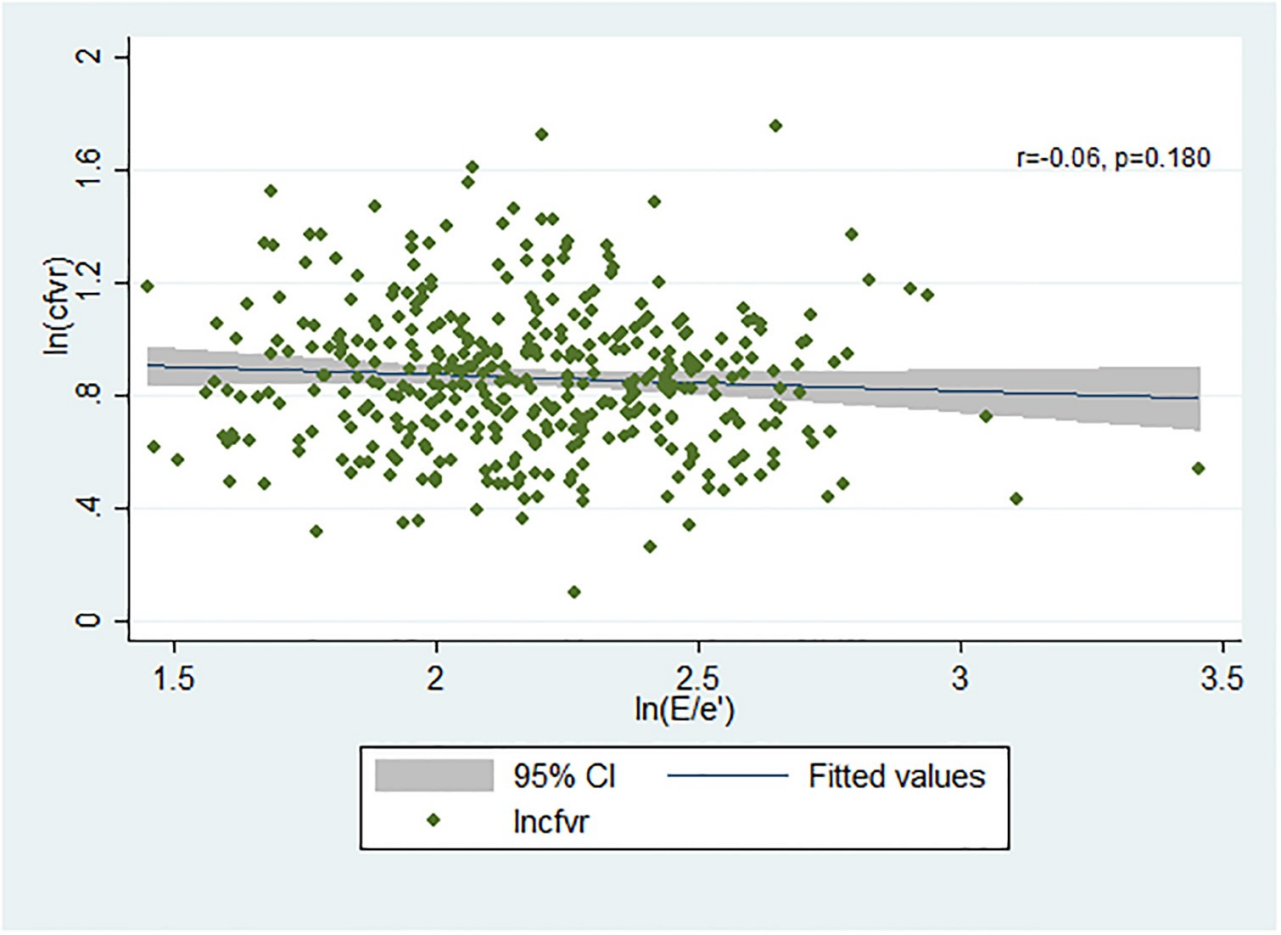
Association between E/e’ and CFVR in the total population. The graph illustrates the effect of E/e’ on CFVR in the total population. Linear regression is performed with both variables naturally log transformed.

We did not find an association between diastolic function measured as E/e’ and CFVR in the overall study population (Fig 3) (referring to the pathway from inflammation through non-endothelial dependent coronary microvascular dysfunction to diastolic dysfunction depicted in Fig 1).

When we restricted the analysis to patients with CFVR<2.5 and E/e’>10 (n = 79) there was a significant association (p = 0.012). Three outliers were found to be influential but they could not be removed after being checked for correctness. However, the association did not reach significance when the outliers were excluded from the regression analysis (p = 0.066). Since the outliers could not be removed and the association therefore reached significance, we proceeded with a mediation analysis. It did not support that the effect of inflammation on diastolic function was mediated by impaired microvascular function in this subpopulation (indirect effect 0.005 (CI -0.011–0.021)), perhaps because of lack of statistical power (only participants with biomarker measurements were included in this analysis (n = 73)). CFVR was associated with other measures of diastolic impairment: E/A (p = 0.013), and LAI (p = 0.039) but not with the combined lateral and medial e’ (p = 0.073) or DECT (p = 0.818).

## 4 Discussion

This is the first study linking data on multiple biomarkers to non-endothelial dependent coronary microvascular function and diastolic function in a large, well-characterized study population. The main finding was that non-endothelial dependent CMD and E/e’ were significantly associated with more than half of the biomarkers studied. Of the 13 biomarkers significantly associated with both CFVR and E/e’, 9 were part of inflammatory pathways, thus providing empirical support to the hypothesis linking inflammation to CMD and diastolic function in patients suspected of microvascular angina as depicted in Fig 1. However, despite a high prevalence of non-endothelial dependent CMD, diastolic dysfunction was not prevalent in the population which questions a direct causal role of non-endothelial dependent CMD in the pathogenesis of diastolic dysfunction and HFpEF.

### 4.1 Non-endothelial CMD and diastolic dysfunction

An underlying hypothesis of the study was that CMD is associated with diastolic dysfunction. The prevalence of CMD was high in the present study, 59% of the patients had CMD defined as CFVR<2.5 and 27% had CFVR<2.0. However, although in particular patients with diabetes had signs of poorer diastolic function, measures were still within a normal range and very few individuals reached the cut-off value indicating diastolic dysfunction (4% among diabetics and none in the other groups). Overall, there was no correlation between CFVR and E/e’, yet we did see an association between CFVR and E/e’ in a subgroup with CMD and E/e’>10. The association, however, was dependent on three influential outliers thus these results should be interpreted with caution and can only be regarded as hypothesis-generating. The data did not directly support a mediating role of non-endothelial CMD in the pathway from inflammation to diastolic function in this subgroup but statistical power was limited and furthermore, it is possible that our study population was too healthy. Importantly, in a prospective study linking non-endothelial dependent coronary microvascular function to development of HFpEF, CFR and E/e’ were inversely correlated only in patients with CFR<2 [23]. In this study of 201 patients without obstructive CAD, E/e’ values were considerably higher than in the present study indicating more advanced stages of disease. In another recent multicenter study of 202 patients diagnosed with HFpEF, the prevalence of CMD was also high [3]. Thus, our results are not contradictory to previous studies but complement them by including also patients with CMD with normal or near-normal diastolic function. Taken together these cross-sectional studies cannot determine whether CMD is a cause or a consequence of diastolic dysfunction but the low prevalence of diastolic dysfunction among patients with impaired microvascular function indicate that non-endothelial dependent CMD is not a strong risk factor for HFpEF. However, our study consisted of a selected population of women in which ethnic origin was not addressed, thus, we cannot rule out that gender differences or race differences may exist, and our results cannot be extrapolated to other patient populations. It is possible that endothelial dependent CMD, which we did not evaluate, is associated with development of diastolic dysfunction. A systemic pro-inflammatory state reduces endothelial nitric oxide synthase activity in coronary endothelial cells, limiting bioavailability for cardiomyocytes. In a preclinical model Schiattarella et al. recently presented evidence that metabolic inflammation and NO synthase are critical in the pathophysiology of HFpEF [27]. MicroRNAs have emerged as another promising tool to identify HFpEF. MicroRNA panels combined with N-terminal pro-B-type natriuretic peptide (NT-proBNP) have shown improved specificity and accuracy in identifying HFpEF in a study of 1710 patients with and without heart failure [28]. Furthermore, microRNAs have shown aetiology-specific transcoronary concentration gradients in heart failure suggesting that microRNAs may be useful biomarkers to distinguish heart failure of different a etiologies [29]. Additionally, preclinical studies have suggested that microRNAs may be released upon ischemia and modulate endothelial function and vascular remodeling on remote vascular beds [30]. Therefore, it may also be a tool to identify early changes in vascular function which awaits further clarification in future clinical studies. In the present study, however, NT-proBNP was not associated with neither CFVR nor E/e’, which is in accordance with previous results suggesting that NT-proBNP level is a better marker in HFrEF [31].

### 4.2 Inflammation

Chronic, subclinical inflammation and its association with cardiovascular disease has been studied extensively in the past years. Previous studies have found increased levels of a number of inflammatory biomarkers in asymptomatic left ventricular systolic- and diastolic dysfunction [32, 33], and inflammatory markers have been associated with increased risk of developing heart failure [34, 35]. Inflammation has also been associated with CMD in several studies [15, 36–40], however, studies were small, study populations selected, and most studies included only a few biomarkers. In this exploratory analysis, out of 90 biomarkers 34 had a significant negative correlation with CFVR, 25 had a significant positive correlation with E/e’, i.e. far more associations than can be explained by chance. Further, the inflammation index was significantly associated with both CFVR and E/e’. These results confirm a link between inflammation and CMD and diastolic dysfunction, respectively, as found in previous studies, and extend them to include a large number of biomarkers that may increase understanding of the pathophysiology behind the conditions.

In line with our findings, several of the biomarkers we found to be associated with both CFVR and E/e’ were also significantly correlated with E/e’ in a prognostic study of 86 patients with HFpEF by Hage et al.: Growth differentiation factor 15 (GDF-15), Tumor necrosis factor receptor 1 (TNFR1), Chitinase-3-like protein 1 (CHI3L1), Fatty acid-binding protein 4 (FABP4) and Soluble urokinase-type plasminogen activator receptor (sUPAR) [20]. In another study TNFR2 levels were significantly associated with increasing E/e’ ratio and grade of diastolic dysfunction in 100 patients with HFpEF, while both TNFR1 and TNFR2 levels were significantly associated with impaired NYHA functional status [21]. In the present study we have found several biomarkers that reflect complex interactions in the cardiovascular system, and the interplay and consequences are not fully understood. In line with this, a recent study found that a combination of biomarker correlations was associated with HFpEF in 431 patients, and that the majority of the biomarkers were related to inflammation [41]. The broad range of biomarkers with involvement in different biological processes may reflect the heterogeneity of both coronary microvascular function and cardiac diastolic function, and it complicates characterization of these entities to a great extent.

### 4.3 Strengths and limitations

To our knowledge this is the largest study exploring an association between a wide range of biomarkers, non-endothelial dependent CMD and cardiac diastolic function. Strengths of the study include the prospective design and that patients were included consecutively among patients undergoing angiography and are therefore less subject to selection bias. However, our study must be considered exploratory due to the large number of examined biomarkers in relation to our population size. The serial analyses performed in this study naturally introduces risk of type I error and the findings should be reproduced in other studies of CMD and as touched upon above, our attempt to achieve extremes of CMD and diastolic function by including patients with diabetes was somewhat undermined by the fact that diastolic dysfunction was less prevalent than expected. We induced hyperemia by dipyridamole infusion which evaluates primarily non-endothelium dependent vasodilatation. Results are not applicable to endothelial dependent coronary vascular function assessed by e.g. acetylcholine provocation.

### 4.4 Conclusions

In this hitherto largest study of cardiovascular biomarkers, coronary microvascular function and cardiac diastolic function we have provided evidence of an association between inflammation and CMD and diastolic function respectively, in a population of women with and without diabetes and with and without angina symptoms. The results suggest that inflammation plays a role in both non-endothelial dependent CMD and diastolic dysfunction, but our data did not support a common pathogenetic pathway between these conditions. This study was performed in a selected patient population and the results cannot be extrapolated to other patient groups. Associations found in this study may be explained by common risk factors and causality must be determined in future studies. Thus, well designed prospective studies are needed to clarify the causality pathway.

## Supporting information

S1 File (DOCX)
