# Supplementary material for: Inflammation, non-endothelial dependent coronary microvascular function and diastolic function—Are they linked?
Source: PLoS One. 2020 Jul 16;15(7):e0236035. doi: 10.1371/journal.pone.0236035 (PMC7365405; doi:10.1371/journal.pone.0236035)
Supplement: S1 File — (DOCX) [file pone.0236035.s001.docx]

**S1: Echocardiographic examination**

We acquired 2-dimensional images of the left ventricle (LV) in apical long axis, 2- and 4-chamber views at frame rates between 60-90 frames/s adjusted as close to the patient’s heart rate as possible. Global longitudinal strain (GLS) was measured using software for speckle tracking analysis (Q-analysis, GE EchoPAC v.112, Norway). Aortic valve closure was defined in tissue Doppler M-mode. GLS was calculated as the average of all accepted segmental values of peak systolic strain (1). Only 3 discarded segments were permitted.

LVEF was analyzed as a semi-automated biplane calculation (Auto-EF tool, GE EchoPAC v.112, Norway). Measurements of left ventricular internal dimensions, left ventricle mass index (LVMI) and left atrium volume index (LAI) (indexed according to body surface area) by the Volume Method of Discs were performed and calculated according to European and American recommendations (2, 3).

Echocardiographic parameters of diastolic function were assessed at rest. Mitral inflow velocities, the E/A ratio, and tissue Doppler of an average of late diastolic velocities in the lateral and septal mitral annulus, e’, were used as surrogate markers of diastolic relaxation and left ventricular compliance; deceleration time as a surrogate of early left ventricular stiffness; and E/e’ as a surrogate estimate of left ventricular filling pressures (4).

Definition of diastolic dysfunction was based on the three criteria: lateral e’<10, E/e’>14 and LAI >34 according to guideline recommendations (4). Tricuspid regurgitation (TR velocity) was not measured since the iPOWER study was planned before the updated recommendation for evaluation of diastolic function in 2016 (5) and a large proportion of the patients had no tricuspid regurgitation. First we calculated whether <50%, 50% or >50% of the above mentioned criteria for diastolic dysfunction were fulfilled. Thus, if all of the three criteria were fulfilled but the TR velocity was missing, diastolic dysfunction was not present. If one criterion was fulfilled, the TR velocity was missing, and two did not reach cut-off it was classified as indeterminate, and if three criteria were fulfilled and the TR velocity was missing, diastolic dysfunction was present. For the indeterminate category we proceeded with an estimation of LV filling pressures by considering the mitral inflow. Thus, if E/A≤0.8 and E≤50cm/s LAP was normal and diastolic dysfunction was graded as I. If E/A≥2, LAP was high suggesting grade III diastolic dysfunction. For those in between with E/A≤0.8 + E≤50cm/s or 0.8<E/A<2, three criteria should be evaluated: average E/e’>14, TR velocity>2.8 m/s and LA vol. index>34 ml/m2. When only two of these were available (no TR velocity measurement), 2 negative corresponded to normal LAP, 2 positive to high LAP and grade II diastolic dysfunction and with 1 positive and 1 negative, LAP and diastolic grade could not be determined. However, classifying participants according to criteria for diastolic dysfunction did not add to the characterization, since very few participants met cut-off values.

For the CFVR examinations two experts, blinded to participant data, analyzed every CFVR examination independently. If estimates differed by >0.2, consensus reading was performed. We have previously reported good inter-analyzer and intra-observer reproducibility (6) as well as good repeatability of CFVR measurements in a patient population (Bland-Altman limits of agreement (CI)=0.44 (0.21;0.68), and correlation coefficient r=0.96 (p<0.01)) and healthy volunteers (limits of agreement (CI)=0.48 (0.22;0.74), r=0.90 (p<0.01)) (7). Before examinations, participants were instructed to be abstinent from caffeine and food containing significant amount of methylxanthine (coffee, tea, chocolate, cola and banana) for 24 hours. Medication containing dipyridamole was paused for 48 hours, anti-ischemic agents (long-lasting nitroglycerine, beta-blockers, calcium antagonist, ivabradine etc.), anti-hypertensive medication and diuretics for 24 hours and short-lasting nitroglycerine for one hour before the examination.

**S2: Biomarker analysis and intensity normalization**

Blood samples were analyzed with the Proximity Extension Assay technique, using the Proseek Multiplex INF 96x96 panel including internal controls. All data were quality controlled and delivered as normalized protein expression (NPX) values, which is an arbitrary, relative quantification unit logarithmically related to protein concentration. Due to the risk of falsely interpreting shifts in median between runs and plates as a biological difference, inter-plate normalization and intensity normalization between batches is necessary. We analyzed samples in two batches, thus intensity normalization was necessary. Results below the limit of detection (LoD) were replaced with the LoD value. Further details regarding LoD, reproducibility and validity are given at http://www.olink.com.

Intensity normalization was performed in the following steps according to recommendations from Olink (8):

1. For each assay and plate, we calculated the plate specific median value.
2. We subtracted the plate specific median for each assay from batch 1 (calculated in step 1) from the median for each assay from batch 2, resulting in the difference between medians for batch 1 and 2 for.
3. Because we wanted to normalize to batch 2, we added the difference in median values between batches (calculated in step 2) to every assay of batch 1.

**S3: Biomarker correlations with CFVR and E/e’ after age-adjustment.**

**Table A**

The table shows standardized regression coefficients with significance level derived from an age-adjusted regression analysis performed with CFVR or E/e' as the dependent variable and each biomarker as predictor variable. *** bold**: biomarkers a priori considered involved in inflammatory response

| **Biomarker (per Z-score)** | **Ln(CFVR)** | **p** | **Ln(E/e')** | **p** |
| --- | --- | --- | --- | --- |
|  | n=395 |  | n=395 |  |
| **13 BIOMARKERS CORRELATED WITH CFVR AND E/e’** |  |  |  |  |
| **Growth/differentiation factor 15 (GDF-15)*** | **-0.049** | **<0.001** | **0.058** | **<0.001** |
| **Soluble urokinase-type plasminogen activator receptor (sU-PAR)*** | **-0.041** | **0.002** | **0.038** | **0.011** |
| **Tumor necrosis factor receptor 1 (TNF-R1)*** | **-0.039** | **0.003** | **0.041** | **0.008** |
| **Peptidoglycan recognition protein 1 (PGLYRP1)*** | **-0.036** | **0.005** | **0.032** | **0.036** |
| Tissue-type plasminogen activator (t-PA) | -0.038 | 0.006 | 0.039 | 0.015 |
| Cathepsin D (CTSD) | -0.034 | 0.009 | 0.045 | 0.003 |
| Cathepsin Z (CTSZ) | -0.034 | 0.010 | 0.044 | 0.003 |
| **Chitinase-3-like protein 1 (CHI3L1)*** | **-0.034** | **0.012** | **0.047** | **0.003** |
| Galectin-4 (Gal-4) | -0.032 | 0.016 | 0.035 | 0.029 |
| **Fatty acid-binding protein, adipocyte (FABP4)*** | **-0.028** | **0.038** | **0.069** | **0.000** |
| **Tumor necrosis factor receptor 2 (TNF-R2)*** | **-0.026** | **0.047** | **0.031** | **0.037** |
| **Retinoic acid receptor responder protein 2 (RARRES2)*** | **-0.026** | **0.050** | **0.039** | **0.010** |
| **Interleukin-2 receptor subunit alpha (IL2-RA)*** | **-0.025** | **0.052** | **0.031** | **0.043** |
| **21 BIOMARKERS CORRELATED WITH CFVR ONLY** |  |  |  |  |
| **Tartrate-resistant acid phosphatase type 5 (TR-AP)*** | **-0.043** | **0.001** | **0.001** | **0.948** |
| C-X-C motif chemokine 16 (CXCL16) | -0.041 | 0.002 | 0.020 | 0.189 |
| **C-C motif chemokine 16 (CCL16)*** | **-0.041** | **0.002** | **0.019** | **0.219** |
| Chitotriosidase-1 (CHIT1) | -0.040 | 0.002 | 0.000 | 0.984 |
| Ephrin type-B receptor 4 (EPHB4) | -0.034 | 0.008 | 0.024 | 0.117 |
| Carboxypeptise A1 (CPA1) | -0.035 | 0.008 | 0.016 | 0.288 |
| Matrix metalloproteinase-9 (MMP-9) | -0.033 | 0.010 | 0.021 | 0.159 |
| **ST2 protein (ST2)*** | **-0.033** | **0.013** | **0.014** | **0.362** |
| Galectin-3 (Gal-3) | -0.032 | 0.015 | 0.010 | 0.527 |
| Interleukin-1 receptor type 2 (IL-1RT2) | -0.031 | 0.016 | -0.001 | 0.928 |
| **Monocyte chemotactic protein 1 (MCP-1)*** | **-0.030** | **0.022** | **0.006** | **0.693** |
| **Interleukin-18-binding protein (IL-18BP)*** | **-0.029** | **0.025** | **0.018** | **0.247** |
| Tumor necrosis factor ligand superfamily member 13B (TNFSF13B) | -0.028 | 0.027 | 0.012 | 0.427 |
| Proprotein convertas subtilisin/kexin type 9 (PCSK9) | -0.029 | 0.027 | 0.007 | 0.636 |
| **TNF receptor superfamily member 10C (TNFRSF10C)*** | **-0.029** | **0.028** | **0.028** | **0.056** |
| Pulmonary surfactant-associated protein D (PSP-D) | -0.029 | 0.032 | -0.021 | 0.166 |
| Carboxypeptidase B (CPB1) | -0.028 | 0.033 | 0.012 | 0.425 |
| Elafin (PI3) | -0.028 | 0.034 | 0.029 | 0.055 |
| **Lymphotoxin-beta receptor (LTBR)*** | **-0.028** | **0.034** | **0.020** | **0.198** |
| **Tumor necrosis facor receptor superfamily member 6 (FAS)*** | **-0.026** | **0.050** | **0.023** | **0.133** |
| Spondin-1 (SPON1) | -0.026 | 0.052 | 0.025 | 0.110 |
| **13 BIOMARKERS CORRELATED WITH E/e’ ONLY** |  |  |  |  |
| Paraoxonase (PON3) | 0.022 | 0.101 | -0.075 | <0.001 |
| **Azurocidin (AZU1)*** | **-0.019** | **0.147** | **0.052** | **0.001** |
| Myeloblastin (PRTN3) | -0.018 | 0.164 | 0.049 | <0.001 |
| Myeloperoxidase (MPO) | -0.015 | 0.236 | 0.054 | <0.001 |
| **E-selectin (SELE)*** | **-0.020** | **0.126** | **0.048** | **0.002** |
| **Transferring receptor protein 1 (TR)*** | **0.006** | **0.661** | **0.043** | **0.005** |
| **C-C motif chemokine 15 (CCL15)*** | **-0.023** | **0.089** | **0.042** | **0.007** |
| **TNF receptor superfamily member 14 (TNFRSF14)*** | **-0.020** | **0.118** | **0.040** | **0.007** |
| **Scavenger receptor cysteine-rich type 1 protein M130 (CD163)*** | **-0.015** | **0.257** | **0.041** | **0.008** |
| Platelet-derived growth factor subunit A (PDGF subunit A) | 0.005 | 0.721 | -0.037 | 0.011 |
| Trefoil factor 3 (TFF3) | -0.025 | 0.059 | 0.034 | 0.021 |
| N-terminal prohormone brain natriuretic peptide (NT-proBNP) | 0.003 | 0.835 | 0.038 | 0.021 |
| Tyrosine-protein phosphatase non-receptor type substrate 1 (SHPS-1) | 0.001 | 0.939 | 0.033 | 0.030 |
| **43 BIOMARKERS NOT CORRELATED WITH CFVR OR E/E’** |  |  |  |  |
| Aminopeptidase (AP-N) | -0.020 | 0.123 | -0.006 | 0.687 |
| Bleomycin hydrolase (BLM hydrolase) | 0.002 | 0.863 | -0.017 | 0.254 |
| Cadherin-5 (CDH5) | -0.010 | 0.464 | -0.009 | 0.539 |
| Caspase-3 (CASP-3) | 0.005 | 0.719 | 0.009 | 0.558 |
| **C-C motif chemokine 24 (CCL24)*** | **-0.013** | **0.321** | **-0.005** | **0.760** |
| CD166 antigen (ALCAM) | -0.016 | 0.225 | 0.010 | 0.506 |
| Collagen alpha-1(I) chain (COL1A1) | 0.002 | 0.875 | -0.011 | 0.465 |
| Complement component C1q receptor (CD93) | -0.019 | 0.148 | 0.007 | 0.643 |
| Contactin-1 (CNTN1) | 0.005 | 0.675 | -0.014 | 0.346 |
| Cystatin-B (CSTB) | -0.025 | 0.056 | 0.021 | 0.146 |
| Epidermal growth factor receptor (EGFR) | 0.001 | 0.922 | -0.014 | 0.350 |
| Granulins (GRN) | -0.022 | 0.094 | 0.026 | 0.093 |
| Insulin-like growth factor-binding protein 1 (IGFBP-1) | 0.000 | 0.991 | -0.025 | 0.103 |
| Insulin-like growth factor-binding protein 2 (IGFBP-2) | -0.001 | 0.882 | -0.023 | 0.143 |
| Insulin-like growth factor-binding protein 7 (IGFBP-7) | -0.018 | 0.176 | 0.016 | 0.303 |
| **Integrin beta-2 (ITGB2)*** | **-0.013** | **0.324** | **0.020** | **0.183** |
| Intercelullar adhesion molecule 2 (ICAM-2) | -0.010 | 0.441 | 0.026 | 0.095 |
| **Interleukin-1 receptor type 1 (IL-1RT1)*** | **-0.021** | **0.108** | **0.019** | **0.201** |
| **Interleukin-17 receptor A (IL-17RA)*** | **-0.018** | **0.177** | **0.005** | **0.733** |
| Interleukin-6 receptor subunit alpha (IL-6RA) | -0.015 | 0.262 | -0.001 | 0.932 |
| **Junctional adhesion molecule a (JAM-A)*** | **-0.003** | **0.787** | **0.017** | **0.276** |
| Kallikrein-6 (KLK6) | -0.017 | 0.191 | 0.002 | 0.916 |
| Low-desity lipoprotein receptor (LDL receptor) | -0.018 | 0.178 | 0.018 | 0.255 |
| Matrix extracellular phosphoglycoprotein (MEPE) | -0.003 | 0.840 | -0.024 | 0.113 |
| Matrix metalloproteinase-2 (MMP-2) | 0.002 | 0.899 | 0.011 | 0.458 |
| Matrix metalloproteinase-3 (MMP-3) | -0.023 | 0.079 | -0.006 | 0.680 |
| Metalloproteinase inhibitor 4 (TIMP4) | -0.021 | 0.111 | 0.007 | 0.653 |
| Myoglobin (MB) | 0.004 | 0.763 | 0.017 | 0.293 |
| Neurogenic locus notch homolog protein 3 (Notch 3) | 0.006 | 0.630 | -0.002 | 0.905 |
| **Osteopontin (OPN)*** | **-0.012** | **0.357** | **0.001** | **0.966** |
| **Osteoprotegerin (OPG)*** | **-0.022** | **0.098** | **0.009** | **0.571** |
| Perlecan (PLC) | -0.011 | 0.389 | 0.020 | 0.196 |
| **Plasminogen activator inhibitor 1 (PAI)*** | **-0.007** | **0.609** | **-0.007** | **0.646** |
| Platelet endothelial cell adhesion molecule (PECAM-1) | 0.006 | 0.648 | 0.008 | 0.603 |
| Protein delta homolog 1 (DLK-1) | 0.000 | 0.977 | -0.003 | 0.858 |
| **P-selectin (SELP)*** | **-0.006** | **0.640** | **0.017** | **0.266** |
| Resistin (RETN) | -0.020 | 0.128 | 0.019 | 0.195 |
| Secretoglobin family 3A member 2 (SCGB3A2) | -0.025 | 0.056 | -0.024 | 0.106 |
| Tissue factor pathway inhibitor (TFPI) | -0.013 | 0.311 | -0.007 | 0.645 |
| Trem-like transcript 2 protein (TLT-2) | 0.001 | 0.949 | 0.019 | 0.199 |
| **Tyrosine-protein kinase receptor UFO (AXL)*** | **-0.002** | **0.886** | **0.009** | **0.537** |
| Urokinase-type plasminogen activator (uPA) | -0.013 | 0.302 | -0.003 | 0.852 |
| Von Willebrand factor (vWF) | -0.014 | 0.269 | 0.005 | 0.763 |

**S4: Level of biomarker across study groups**

**Table A**

|  | **iPOWER cohort** |  |  | **Copenhagen City**  **Heart Study cohort** |  |  |
| --- | --- | --- | --- | --- | --- | --- |
|  | **Group A** | **Group B** |  | **Group C** |  |  |
| **Biomarker (mean (SD))** | **Diabetes**  **(n=159)** | **No diabetes**  **(n=156)** | **P^#^** | **Control**  **(n=80)** | **P^#^** | **P** |
| Aminopeptidase (AP-N) | 6.14 (0.35) | 6.06 (0.50) | 0.102 | 5.98 (0.38) | 0.008 | **0.007**† |
| Azurocidin (AZU1)* | 8.09 (1.21) | 7.18 (1.08) | <0.001 | 7.83 (0.99) | 0.072 | **0.002**† |
| Bleomycin hydrolase (BLM hydrolase) | 6.43 (0.55) | 6.41 (0.62) | 0.944 | 6.39 (0.45) | 0.815 | 0.849 |
| Cadherin-5 (CDH5) | 4.98 (0.41) | 5.09 (0.61) | 0.025 | 4.88 (0.45) | 0.251 | 0.620 |
| Carboxypeptise A1 (CPA1) | 7.30 (0.88) | 7.15 (0.64) | 0.119 | 7.09 (0.62) | 0.069 | **0.049**† |
| Carboxypeptidase B (CPB1) | 6.93 (0.81) | 6.83 (0.61) | 0.320 | 6.70 (0.57) | 0.038 | **0.039**† |
| Caspase-3 (CASP-3) | 6.82 (1.26) | 6.34 (1.14) | <0.001 | 6.91 (1.23) | 0.693 | 0.638 |
| Cathepsin D (CTSD) | 5.91 (0.58) | 5.52 (0.61) | <0.001 | 5.52 (0.51) | <0.001 | **<0.001**† |
| Cathepsin Z (CTSZ) | 6.47 (0.45) | 6.15 (0.59) | <0.001 | 6.12 (0.43) | <0.001 | **<0.001**† |
| C-C motif chemokine 15 (CCL15)* | 8.79 (0.61) | 8.21 (0.71) | <0.001 | 8.49 (0.70) | 0.006 | **<0.001**† |
| C-C motif chemokine 16 (CCL16)* | 7.90 (0.68) | 7.57 (0.62) | <0.001 | 7.49 (0.64) | <0.001 | **<0.001**† |
| C-C motif chemokine 24 (CCL24)* | 6.88 (0.93) | 6.83 (0.97) | 0.749 | 6.76 (0.91) | 0.423 | 0.435 |
| CD166 antigen (ALCAM) | 6.81 (0.35) | 6.83 (0.50) | 0.433 | 6.66 (0.39) | 0.020 | 0.062 |
| Chitinase-3-like protein 1 (CHI3L1)* | 8.47 (1.04) | 7.91 0.920) | <0.001 | 7.96 (0.90) | 0.001 | **<0.001**† |
| Chitotriosidase-1 (CHIT1) | 7.90 (1.17) | 7.65 (1.76) | 0.286 | 7.56 (1.65) | 0.229 | 0.191 |
| Collagen alpha-1(I) chain (COL1A1) | 3.90 (0.46) | 3.94 (0.53) | 0.420 | 3.99 (0.47) | 0.152 | 0.147 |
| Complement component C1q receptor (CD93) | 12.26 (0.36) | 12.32 (0.49) | 0.116 | 12.20 (0.39) | 0.480 | 0.792 |
| Contactin-1 (CNTN1) | 5.45 (0.38) | 5.50 (0.50) | 0.239 | 5.42 (0.41) | 0.841 | 0.927 |
| C-X-C motif chemokine 16 (CXCL16) | 6.58 (0.37) | 6.50 (0.52) | 0.131 | 6.43 (0.41) | 0.018 | **0.015**† |
| Cystatin-B (CSTB) | 6.42 (0.79) | 6.07 (0.78) | <0.001 | 5.93 (0.58) | <0.001 | **<0.001**† |
| Elafin (PI3) | 5.02 (0.69) | 4.46 (0.71) | <0.001 | 4.73 (0.60) | 0.003 | **<0.001**† |
| Ephrin type-B receptor 4 (EPHB4) | 5.63 (0.41) | 5.56 (0.38) | 0.170 | 5.50 (0.37) | 0.030 | **0.026**† |
| Epidermal growth factor receptor (EGFR) | 4.10 (0.28) | 4.15 (0.40) | 0.265 | 4.09 (0.36) | 0.727 | 0.957 |
| E-selectin (SELE)* | 14.07 (0.72) | 13.78 (0.57) | <0.001 | 13.64 (0.63) | <0.001 | **<0.001**† |
| Fatty acid-binding protein, adipocyte (FABP4)* | 7.91 (0.90) | 7.23 (0.82) | <0.001 | 7.09 (0.69) | <0.001 | **<0.001**† |
| Galectin-3 (Gal-3) | 7.36 (0.56) | 7.17 (0.60) | 0.011 | 7.00 (0.47) | <0.001 | **<0.001**† |
| Galectin-4 (Gal-4) | 5.32 (0.68) | 4.71 (0.62) | <0.001 | 4.69 (0.57) | <0.001 | **<0.001**† |
| Granulins (GRN) | 7.65 (0.37) | 7.45 (0.48) | <0.001 | 7.44 (0.40) | 0.001 | **<0.001**† |
| Growth/differentiation factor 15 (GDF-15)* | 7.46 (0.89) | 6.74 (0.65) | <0.001 | 6.71 (0.60) | <0.001 | **<0.001**† |
| Insulin-like growth factor-binding protein 1 (IGFBP-1) | 6.11 (1.17) | 6.13 (0.94) | 0.723 | 6.34 (0.79) | 0.069 | 0.091 |
| Insulin-like growth factor-binding protein 2 (IGFBP-2) | 8.78 (0.68) | 8.90 (0.69) | 0.042 | 8.91 (0.64) | 0.058 | **0.032** |
| Insulin-like growth factor-binding protein 7 (IGFBP-7) | 9.00 (0.56) | 8.88 (0.57) | 0.161 | 8.76 (0.47) | 0.007 | **0.007**† |
| Integrin beta-2 (ITGB2)* | 6.89 (0.84) | 6.66 (0.49) | 0.005 | 6.63 (0.78) | 0.007 | **0.003**† |
| Intercelullar adhesion molecule 2 (ICAM-2) | 6.29 (0.43) | 6.13 (0.58) | 0.010 | 6.11 (0.45) | 0.012 | **0.005**† |
| Interleukin-1 receptor type 1 (IL-1RT1)* | 7.53 (0.44) | 7.42 (0.49) | 0.043 | 7.33 (0.39) | 0.003 | **0.002**† |
| Interleukin-1 receptor type 2 (IL-1RT2) | 6.21 (0.47) | 6.27 (0.50) | 0.225 | 6.13 (0.40) | 0.227 | 0.431 |
| Interleukin-17 receptor A (IL-17RA)* | 4.99 (0.61) | 5.10 (0.63) | 0.105 | 4.98 (0.57) | 0.979 | 0.729 |
| Interleukin-18-binding protein (IL-18BP)* | 7.36 (0.45) | 7.30 (0.55) | 0.530 | 7.14 (0.47) | 0.005 | **0.008**† |
| Interleukin-2 receptor subunit alpha (IL2-RA)* | 5.34 (0.54) | 5.18 (0.60) | 0.040 | 5.14 (0.59) | 0.030 | **0.017**† |
| Interleukin-6 receptor subunit alpha (IL-6RA)* | 13.49 (0.40) | 13.46 (0.56) | 0.640 | 13.38 (0.47) | 0.146 | 0.164 |
| Junctional adhesion molecule a (JAM-A)* | 6.56 (0.84) | 6.25 (0.62) | 0.001 | 6.71 (0.99) | 0.188 | 0.703 |
| Kallikrein-6 (KLK6) | 6.78 (0.40) | 6.69 0.481) | 0.092 | 6.74 (0.38) | 0.574 | 0.378 |
| Low-desity lipoprotein receptor (LDL receptor) | 6.50 (0.75) | 6.35 (0.68) | 0.128 | 6.15 (0.65) | 0.001 | **0.001**† |
| Lymphotoxin-beta receptor (LTBR)* | 5.31 (0.46) | 5.21 (0.48) | 0.072 | 5.13 (0.38) | 0.008 | **0.006**† |
| Matrix extracellular phosphoglycoprotein (MEPE) | 6.51 (0.59) | 6.65 (0.53) | 0.038 | 6.63 (0.54) | 0.128 | 0.070 |
| Matrix metalloproteinase-2 (MMP-2) | 4.62 (0.43) | 4.58 (0.56) | 0.653 | 4.59 (0.43) | 0.985 | 0.905 |
| Matrix metalloproteinase-3 (MMP-3) | 7.19 (0.54) | 7.20 (0.67) | 0.570 | 7.15 (0.53) | 0.963 | 0.929 |
| Matrix metalloproteinase-9 (MMP-9) | 7.48 (0.65) | 7.17 (0.71) | <0.001 | 7.26 (0.60) | 0.011 | **0.002**† |
| Metalloproteinase inhibitor 4 (TIMP4) | 4.66 (0.53) | 4.62 (0.69) | 0.987 | 4.58 (0.57) | 0.743 | 0.769 |
| Monocyte chemotactic protein 1 (MCP-1)* | 6.04 (0.51) | 5.94 (0.51) | 0.142 | 5.88 (0.49) | 0.050 | **0.038**† |
| Myeloblastin (PRTN3) | 7.58 (1.15) | 6.75 (0.90) | <0.001 | 7.28 (1.00) | 0.014 | **<0.001**† |
| Myeloperoxidase (MPO) | 5.84 (0.66) | 5.25 (0.71) | <0.001 | 5.73 (0.61) | 0.200 | **0.006**† |
| Myoglobin (MB) | 7.51 (0.63) | 7.43 (0.66) | 0.743 | 7.47 (0.51) | 0.714 | 0.803 |
| Neurogenic locus notch homolog protein 3 (Notch 3) | 5.85 (0.50) | 5.92 (0.59) | 0.075 | 5.91 (0.43) | 0.130 | 0.081 |
| N-terminal prohormone brain natriuretic peptide (NT-proBNP) | 5.17 (1.21) | 5.18 (0.48) | 0.407 | 5.14 (0.84) | 0.638 | 0.545 |
| Osteopontin (OPN)* | 7.56 (0.62) | 7.67 (0.65) | 0.038 | 7.50 (0.55) | 0.814 | 0.791 |
| Osteoprotegerin (OPG)* | 5.41 (0.64) | 5.25 (0.51) | 0.054 | 5.17 (0.46) | 0.009 | **0.006**† |
| Paraoxonase (PON3) | 5.97 (0.68) | 6.77 (0.73) | <0.001 | 6.47 (0.71) | <0.001 | **<0.001** |
| Peptidoglycan recognition protein 1 (PGLYRP1)* | 9.52 (0.66) | 9.21 (0.70) | <0.001 | 9.30 (0.59) | 0.015 | **0.002**† |
| Perlecan (PLC) | 8.29 (0.74) | 7.99 (0.48) | <0.001 | 8.07 (0.67) | 0.028 | **0.005**† |
| Plasminogen activator inhibitor 1 (PAI)* | 8.46 (0.57) | 8.33 (0.59) | 0.030 | 8.50 (0.46) | 0.683 | 0.894 |
| Platelet endothelial cell adhesion molecule (PECAM-1) | 5.74 (0.59) | 5.59 (0.53) | 0.031 | 5.90 (0.73) | 0.042 | 0.198 |
| Platelet-derived growth factor subunit A (PDGF subunit A) | 7.70 (0.74) | 7.82 (0.66) | 0.124 | 7.81 (0.52) | 0.251 | 0.173 |
| Proprotein convertas subtilisin/kexin type 9 (PCSK9) | 3.69 (0.46) | 3.54 (0.43) | 0.007 | 3.40 (0.44) | <0.001 | **<0.001**† |
| Protein delta homolog 1 (DLK-1) | 7.54 (0.67) | 7.55 (0.71) | 0.420 | 7.46 (0.61) | 0.773 | 0.944 |
| P-selectin (SELP)* | 11.76 (0.57) | 11.53 (0.72) | 0.003 | 11.83 (0.61) | 0.418 | 0.980 |
| Pulmonary surfactant-associated protein D (PSP-D) | 3.99 (0.90) | 4.13 (0.66) | 0.034 | 3.76 (0.77) | 0.115 | 0.369 |
| Resistin (RETN) | 7.95 (0.76) | 7.71 (0.62) | 0.001 | 7.76 (0.61) | 0.035 | **0.010**† |
| Retinoic acid receptor responder protein 2 (RARRES2)* | 12.76 (0.29) | 12.49 (0.43) | <0.001 | 12.60 (0.34) | 0.002 | **<0.001**† |
| Scavenger receptor cysteine-rich type 1 protein M130 (CD163)* | 8.79 (0.54) | 8.62 (0.61) | 0.017 | 8.50 (0.49) | <0.001 | **<0.001**† |
| Secretoglobin family 3A member 2 (SCGB3A2) | 3.78 (0.85) | 4.20 (1.10) | <0.001 | 3.94 (0.84) | 0.187 | **0.041** |
| Soluble urokinase-type plasminogen activator receptor (sU-PAR)* | 7.19 (0.58) | 6.86 (0.58) | <0.001 | 6.98 (0.56) | 0.012 | **0.001**† |
| Spondin-1 (SPON1) | 1.85 (0.44) | 1.78 (0.38) | 0.270 | 1.64 (0.33) | 0.001 | **0.001**† |
| ST2 protein (ST2)* | 5.36 (0.59) | 5.12 (0.61) | 0.001 | 5.14 (0.51) | 0.021 | **0.006**† |
| Tartrate-resistant acid phosphatase type 5 (TR-AP)* | 5.59 (0.43) | 5.45 (0.52) | 0.039 | 5.23 (0.48) | <0.001 | **<0.001**† |
| Tissue factor pathway inhibitor (TFPI) | 10.81 (0.52) | 10.82 (0.57) | 0.657 | 10.76 (0.49) | 0.727 | 0.836 |
| Tissue-type plasminogen activator (t-PA) | 8.57 (0.94) | 8.09 (0.72) | <0.001 | 8.05 (0.79) | <0.001 | **<0.001**† |
| Transferring receptor protein 1 (TR)* | 5.15 (0.64) | 4.79 (0.63) | <0.001 | 4.85 (0.69) | 0.001 | **<0.001**† |
| Trefoil factor 3 (TFF3) | 6.87 (0.69) | 6.51 (0.71) | <0.001 | 6.52 (0.51) | <0.001 | **<0.001**† |
| Trem-like transcript 2 protein (TLT-2) | 6.44 (0.51) | 6.36 (0.60) | 0.183 | 6.50 (0.53) | 0.489 | 0.759 |
| Tumor necrosis factor ligand superfamily member 13B (TNFSF13B) | 8.45 (0.44) | 8.42 (0.52) | 0.671 | 8.26 (0.40) | 0.006 | **0.012**† |
| Tumor necrosis factor receptor 1 (TNF-R1)* | 7.93 (0.48) | 7.74 (0.50) | 0.002 | 7.65 0.451) | <0.001 | **<0.001**† |
| Tumor necrosis factor receptor 2 (TNF-R2)* | 6.62 (0.71) | 6.30 (0.69) | <0.001 | 6.26 (0.47) | <0.001 | **<0.001**† |
| Tumor necrosis factor receptor superfamily member 10C (TNFRSF10C)* | 7.83 (0.61) | 7.63 (0.68) | 0.005 | 7.60 (0.57) | 0.010 | **0.004**† |
| Tumor necrosis factor receptor superfamily member 14 (TNFRSF14)* | 6.42 (0.48) | 6.12 (0.58) | <0.001 | 6.37 (0.44) | 0.614 | 0.130 |
| Tumor necrosis facor receptor superfamily member 6 (FAS)* | 6.76 (0.46) | 6.61 (0.50) | 0.023 | 6.57 (0.45) | 0.012 | **0.006**† |
| Tyrosine-protein kinase receptor UFO (AXL)* | 10.01 (0.38) | 9.93 (0.53) | 0.168 | 9.83 (0.44) | 0.007 | **0.007**† |
| Tyrosine-protein phosphatase non-receptor type substrate 1 (SHPS-1) | 4.69 (0.50) | 4.54 (0.58) | 0.017 | 4.56 (0.47) | 0.115 | **0.053**† |
| Urokinase-type plasminogen activator (uPA) | 6.86 (0.61) | 6.82 (0.48) | 0.510 | 6.79 (0.51) | 0.343 | 0.322 |
| Von Willebrand factor (vWF) | 9.51 (0.80) | 9.17 (1.06) | 0.003 | 9.23 (0.89) | 0.054 | **0.017**† |

Level of biomarker as mean normalized protein expression value with standard deviation compared across groups with trend test (age adjusted linear regression). *Biomarkers with involvement in an inflammatory response according to Olink Proteomics (8). ^#^compared with group A. †significant negative trend across groups A-C. P for trend <0.05 highlighted in bold.

**References**

1. Voigt JU, Pedrizzetti G, Lysyansky P, Marwick TH, Houle H, Baumann R, et al. Definitions for a common standard for 2D speckle tracking echocardiography: consensus document of the EACVI/ASE/Industry Task Force to standardize deformation imaging. J Am Soc Echocardiogr. 2015;28(2):183-93.

2. Lang RM, Badano LP, Mor-Avi V, Afilalo J, Armstrong A, Ernande L, et al. Recommendations for cardiac chamber quantification by echocardiography in adults: an update from the American Society of Echocardiography and the European Association of Cardiovascular Imaging. Eur Heart J Cardiovasc Imaging. 2015;16(3):233-70.

3. Barbieri A, Bursi F, Mantovani F, Valenti C, Quaglia M, Berti E, et al. Left ventricular hypertrophy reclassification and death: application of the Recommendation of the American Society of Echocardiography/European Association of Echocardiography. Eur Heart J Cardiovasc Imaging. 2012;13(1):109-17.

4. Nagueh SF, Smiseth OA, Appleton CP, Byrd BF, 3rd, Dokainish H, Edvardsen T, et al. Recommendations for the Evaluation of Left Ventricular Diastolic Function by Echocardiography: An Update from the American Society of Echocardiography and the European Association of Cardiovascular Imaging. Eur Heart J Cardiovasc Imaging. 2016;17(12):1321-60.

5. Nagueh SF, Appleton CP, Gillebert TC, Marino PN, Oh JK, Smiseth OA, et al. Recommendations for the evaluation of left ventricular diastolic function by echocardiography. Eur J Echocardiogr. 2009;10(2):165-93.

6. Prescott E, Abildstrom SZ, Aziz A, Merz NB, Gustafsson I, Halcox J, et al. Improving diagnosis and treatment of women with angina pectoris and microvascular disease: the iPOWER study design and rationale. Am Heart J. 2014;167(4):452-8.

7. Michelsen MM, Mygind ND, Pena A, Olsen RH, Christensen TE, Ghotbi AA, et al. Transthoracic Doppler echocardiography compared with positron emission tomography for assessment of coronary microvascular dysfunction: The iPOWER study. Int J Cardiol. 2017;228:435-43.

8. <http://www.olink.com>. Olink Proteomics. 2018.
